# The impact of extreme El Niño events on modern sediment transport along the western Peruvian Andes (1968–2012)

**DOI:** 10.1038/s41598-017-12220-x

**Published:** 2017-09-25

**Authors:** Sergio B. Morera, Thomas Condom, Alain Crave, Philippe Steer, Jean L. Guyot

**Affiliations:** 1 Instituto Geofísico del Perú, Lima, 15012 Peru; 2 Universidad Nacional Mayor de San Marcos, Lima, 15081 Peru; 3 Université de Grenoble Alpes, CNRS, IRD, IGE, F-38000 Grenoble, France; 4 UMR 6118 Géosciences Rennes, Université Rennes 1, CNRS, Rennes, 35043 France; 5 Université de Toulouse, CNRS, GET, IRD, Lima, 15036 Peru

## Abstract

Climate change is considered as one of the main factors controlling sediment fluxes in mountain belts. However, the effect of El Niño, which represents the primary cause of inter-annual climate variability in the South Pacific, on river erosion and sediment transport in the Western Andes remains unclear. Using an unpublished dataset of Suspended Sediment Yield (SSY) in Peru (1968–2012), we show that the annual SSY increases by 3–60 times during Extreme El Niño Events (EENE) compared to normal years. During EENE, 82% to 97% of the annual SSY occurs from January to April. We explain this effect by a sharp increase in river water discharge due to high precipitation rates and transport capacity during EENE. Indeed, sediments accumulate in the mountain and piedmont areas during dry normal years, and are then rapidly mobilized during EENE years. The effect of EENE on SSY depends on the topography, as it is maximum for catchments located in the North of Peru (3–7°S), exhibiting a concave up hypsometric curve, and minimum for catchments in the South (7–18°S), with a concave down hypsometric curve. These findings highlight how the sediment transport of different topographies can respond in very different ways to large climate variability.

## Introduction

Numerous studies on erosion mass balance at global, regional or local scales highlight the control of geological^1,2^, climatic^3,4^ and anthropical^5–7^ constrains on erosion rates. Indeed, some of the highest recorded erosion rates occur in tropical mountain ranges, where large magnitude earthquakes and rainfall events are frequent^8,9^. This observation questions the impact of such extreme events, relatively to average conditions, on the source to sink mass balance of mountains belts. This is particularly timely since the current global climate change may affect the relative frequencies of large and small rainfall events in many places, at local or regional scales^9^. Because of its large range of climatic conditions and its rainfall variability, the west coast of the Andes represents one of the most appropriate mountain range to investigate this issue. In particular, the El Niño–Southern Oscillation (ENSO) induces a large spatial variability of annual precipitation from North to South and along the coast. Exploring the impact of El Niño on the sediment discharge at the outlet of the western Andean basins can help to decipher the rainfall characteristics that control the historical and long-term trend of sediment fluxes.

The Andes with ~9000 km of mountain chains cover a wide range of climate zones, from the dry Sechura and Atacama deserts to the humid Amazon basin. The western Peruvian Andes (3–18°S) are located in a region where frequent floods and recurrent large earthquakes occur^10^, which in turn influence sediment fluxes in this mountainous region^11,12^. It is also an area where extreme hydrological and climatological processes occur, partly driven by the irregularly periodical ENSO, which is characterized by changes in atmospheric pressure and sea surface temperature around the Pacific Ocean. When an El Niño or a La Niña event dominates the Southern Oscillation (SO), exceptionally warm or cold ocean temperatures occur in the tropical Pacific, respectively^13^. As a result, El Niño and La Niña have significant impacts on the climate in the Andes and its spatial and temporal variability.

Throughout Andean human history, El Niño events have caused catastrophic cultural destruction^14^, and the periods September 1982 to August 1983 and September 1997 to August 1998 are categorized as the strongest meteorological events since the last ice age in the early Holocene epoch^15^. Quantitative elements based on ENSO indices allows to define them as “Extreme El Niño Events” (EENE), that have a strong impact on ecosystems, agriculture, and extreme weather events at global scale^16,17^. Over the past 35 years, EENE have affected the climatology, hydrology and also the sedimentology of catchments all along the Andes, yet with an impact that varies spatially. In western Ecuador (1°N–3°S)^18^ and northwestern Peru (3–5°S)^19^, EENE produce 5.4–11 times the mean river discharge (Q) and mean suspended sediment yield (SSY), respectively. Consequently, farmlands, houses, bridges, and roads are destroyed, which causes great economic losses, and floods and epidemics kill thousands of people and animals^20^. In central Chile (27–35°S), an increase in runoff and SSY by a factor of two were observed^21^. In contrast, dry conditions or low Q and SSY were observed in southern Chile (35–40°S)^22^, the eastern Bolivian Andes^23^, northwestern Colombia (2–11°N)^24^ and northwestern Venezuela^25^. Western Peru, characterized by a large range of different landscapes, topographies and climates, is therefore a key area to understand the impact of EENE on sediment transport. In addition, although high mountainous systems, i.e. areas above 1000 m sea level^26^, on the western side cover only 7% of Peru, they supply fresh water to 64% of the 31 million inhabitants. To benefit from this natural resource, successive national governments have invested billions of dollars in building eight multipurpose hydraulic systems along the Peruvian coast for fresh water use. However, the high suspended sediment load occurring during EENE places water infrastructures and water availability at high risk, which intensifies local conflicts for access to water and affects aquatic ecosystems. Additionally, some studies suggest that the recurrence and severity of the EENE will increase during the global warming and climate changes^17,27^.

Nevertheless, up to now, no complete or robust dataset existed on sediment transport for western Peru^28^. Indeed, no single institution monitors, processes, and supplies a national hydro-sedimentology database using consistent methods. Therefore, records are often incomplete and not widely available, in turn preventing a clear and robust assessment of sediment transfer in Peru from the central Andes to the coastal plane and to the Pacific Ocean. Thus, it is still unknown how EENE impacts the hydrosedimentary processes and hazards involved during sediment transfer from the continent to the ocean. In response, this study compiles an unpublished and extensive database of SSY in Peru and investigates whether EENE is the main agent responsible for the spatial and temporal variability in SSY along the western Andes.

## Materials and Methods

### Geomorphological setting

**Figure 1:**
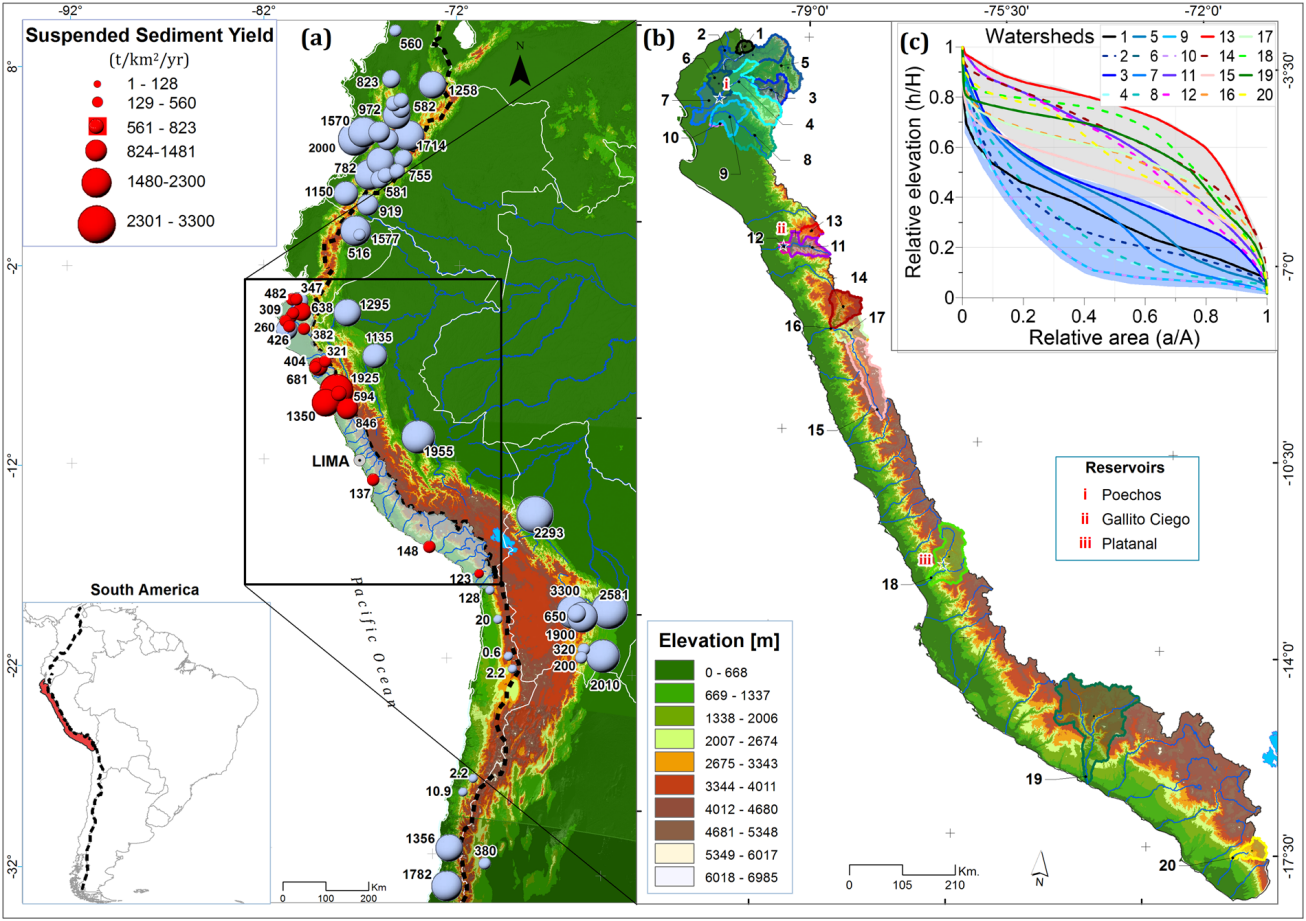
(**a**) Suspended sediment yield (SSY) data along the Andes Mountains. Circle centres indicate the locations of hydro-sedimentological stations, while their size is proportional to the SSY. Red and lead circles represent data from this study and the literature^29–38^, respectively. The black dotted line separates the Pacific and Atlantic watersheds. (**b**) Location of the 20 studied stations/watersheds in the northern, central and southern Peruvian Andes. Watersheds are numbered generally from north to south, according to Table 1. Topography is based on the Shuttle Radar Topography Mission (SRTM) digital elevation model (DEM) with a 90 m × 90 m resolution. (**c**) Studied catchments with concave up (blue background) or concave down (gray background) normalized hypsometric curves are located in North (3–7°S) and South (7–18°S) Peru, respectively. The map was generated using ArcGIS for Desktop 9.3 (www.esri.com/software/arcgis).

The western Peruvian Andes (study area) are located on the Pacific coast from 3–18°S and 70–81°E. They run from Ecuador to Chile and are bounded in the east by the Amazon watershed and in the west by the Pacific Ocean. Geology and geomorphology of Peru is strongly associated with the Andean orogeny that began during the early Cretaceous period and took its present form during the Cenozoic era. In turn, Andean watersheds have a wide variety of underlying lithology. In this study, 20 hydrologic stations and watersheds, with a surface area ranging from 638 to 16,949 km^2^, were studied along the western Peruvian coast (Fig. 1).

**Table 1:** Locations details, watershed topographies, and geomorphologic characteristics of hydro-sedimentology stations.

Stations											Watershed				
Code	Watershed	Station	Coordinates (deg.)		Altitude (m)	Q (period)	Q (n)	SSC (period)	SSC (n)	SSC Gaps	Elevation (m)		Area (km^2^)	Avg. Slope (degree)	Treatment.
			Lon.	Lat.							Avg.	Max.			
1	Zarumilla	Palmales**	−80.19	−3.65	42	1980–2012	3428	2011–2012	86	71%	319	1242	639	7.5	a, b, c
2	Tumbes	El Tigre**	−80.47	−3.78	22	1963–2012	17897	2004–2012	2082	13%	1023	3875	4708	16.2	a, b, c
3	Chira	Macara*	−79.94	−4.41	384	1973–2012	14244	1973–1992	5896	14%	1745	3866	2472	19.4	a, b, c
4	Chira	Ardilla**	−80.42	−4.50	105	1976–2012	13148	1980–1992	1926	52%	674	3048	11934	9.1	a, c
5	Chira	Ciruelo*	−80.13	−4.27	228	1975–2012	13515	—	—	—	1756	3772	4230	20.2	a, c
6	Chira	Aliviadero**	−80.52	−4.67	62	1976–2012	13148	1981–1992	3955	10%	1406	3946	13546	16.4	a, c
7	Chira	Pte. Sullana**	−80.82	−4.89	16	1972–2012	14609	1972–1992	7094	13%	1210	3946	16949	14.1	a, b, c
8	Piura	Pte Ñacara**	−80.19	−5.12	81	1972–2012	14609	1973–1974	33	95%	876	3656	4662	14.6	a, b, c
9	Piura	Ejidos**	−80.63	−5.13	24	1986–2012	9497	1986–2006	6136	15%	629	3656	7490	11.1	a, b, c
10	Piura	Sanch Cerr**	−80.62	−5.17	22	1972–2002	12428	1973–1992	5462	21%	623	3656	7614	10.5	a, b, c
11	PEJEZA	Yonan*	−79.09	−7.24	441	1986–2012	9497	2003–2012	2964	10%	2571	4195	3290	19.5	a, b, c
12	PEJEZA	Ventanilla*	−79.27	−7.29	246	1949–2012	9252	1968–1975	2556	12%	2411	4195	3631	19.4	a, c
13	PEJEZA	Paltas*	−78.90	−7.18	752	1970–2012	1674	2007–2012	1673	24%	3101	4158	1035	14.4	a, c
14	Santa	Tablachaca*	−78.23	−8.65	525	2002–2012	3609	2002–2012	3542	12%	3270	4969	3131	20.4	a, b, c
15	Santa	La Balsa*	−77.83	−8.86	1833	1954–2012	17897	1975–2007	8182	30%	4061	6685	4933	19.3	a, b, c
16	Santa	Condorcerro*	−78.26	−8.66	470	1957–2012	16709	1999–2012	4726	9%	3655	6685	10403	21.1	a, c
17	Santa	Santa*	−78.21	−8.66	523	2002–2012	3653	2002–2012	3652	11%	3827	6685	7190	21.3	a, b, c
18	Cañete	Socsi*	−76.21	−13.03	310	1974–2012	13879	2004–2012	182	93%	3778	5873	5803	22.4	a, b, c
19	Ocoña	Ocoña**	−73.15	−16.39	15	1984–2012	3657	2007–2011	126	91%	3764	6403	15971	15.1	a, b, c
20	Sama	Sambalay*	−70.48	−17.62	924	1963–2012	17897	1997–2012	439	82%	3541	5719	1701	17.5	a, b, c

Q = daily average discharge, SSC = daily average suspended sediment concentration. Q(n) and SSC(n) are the numbers of daily Q and SSC measurements, respectively. ^a^Hydro-sedimentology inconsistencies data analysis. ^b^Fill SSY daily gaps.

^c^Frequency sampling and uncertainty analysis. *Mountain station. **Piedmont station.

Eleven stations are located in the Andean mountains (228–1833 m a.s.l.), and nine are located downstream in the piedmont (15–105 m a.s.l.) (Table 1). Most monitored rivers flow perpendicular to the Andean chain and drain into the western escarpment. Main rivers begin at 2800–5000 m a.s.l. and range from 80–550 km in length^39^. The study area can be divided into three latitudinal regions: (i) northern, which has a low local relief and elevations below 4500 m a.s.l.; (ii) central, which has steep slopes and a rugged topography reaching 6768 m a.s.l., with more than 15 peaks higher than 6000 m a.s.l.^40^; and (iii) southern, which rises up to ∼6000 m a.s.l. and includes steep rivers. This spatial variability of the topography is also associated to marked differences in the hypsometry of the considered catchments with: catchments in northern Peru that have concave up hypsometric curves, while catchments in central and northern Peru have concave down hypsometric curves (Fig. 1; section Dataset and Data Analyst).

### Climate and Extreme El Niño events in the western Peruvian Andes

Because the Andes form the main barrier to atmospheric circulation in the Southern Hemisphere^41^, they strongly influence the global and South American climates. ENSO strongly alters the inter-annual variability of climate and hydrology in the Andes. Its spatiotemporal characteristics involve distinct phases. In recent decades, study of ENSO has produced several classifications, such as weak, moderate and strong El Niño/La Niña, as well as EENE, canonical and Modoki El Niño e.g.^16,42^. The intensity of the ENSO phase and its impact on the Pacific coast remain under debate. Dozens of El Niño/La Niña indexes exist and depend greatly on the parameter and/or the region of the ocean considered, e.g. El Niño 1 + 2, 3, 3.4, and 4. In this study we only focus on two EENE periods (1982–1983 and 1997–1998), which are two of the most outstanding events that have had consequences at the worldwide scale e.g.^42^. Using quantitative arguments to define EENE, an El Niño event is considered extreme when more than 9 consecutive months have indices E (Eastern Pacific) of the ENSO^16^ higher than two. This occurs only in 1982/1983 and 1997/1998 over the monitored time period [1963–2012]. Therefore, we differentiate two climatic responses based on their impact on the Pacific coast of Peru. The first are EENE periods, which include two periods, from 1 September 1982 to 31 August 1983 and from 1 September 1997 to 31 August 1998. The second are non-EENE periods, which include normal years and weak, moderate and strong El Niño/La Niña variants.

During normal years, mean annual precipitation in the western Peruvian Andes is generally low and displays a latitudinal gradient (~1000 mm/yr in the north to ~400 mm/yr in the south). The hydrological year begins in September and ends in August in all watersheds and does not change with latitude. Seasonal expansion of easterly equatorial winds carry moisture from the Atlantic to the Andes and cause precipitation to occur mainly during the austral summer (January-March)^43^. On average, 90% of annual precipitation falls from October-April, with a peak in February and March. The rest of the year (May to September) is dry, with less than 50–100 mm of precipitation^44^.

During EENE, a pronounced eastward extension of the western Pacific warms and develops atmospheric convection, which increases atmospheric moisture and greatly increases rainfall in the usually cold and dry equatorial eastern Pacific. This massive reorganization of atmospheric convection causes severe disturbances in global weather patterns e.g.^17,27^. In Peru, daily precipitation is more frequent and stronger during EENE years than during normal years^45^. Three sub-regions with different precipitation patterns during EENE were observed in northern, central and southern Peru^46^. The most pronounced effect of EENE on precipitation occurs in Northern Peru, which experiences an easterly wind-driven monsoon in the mountains and intermittent westerly wind-driven intense precipitation events on the Pacific coast. Consequently, catastrophic floods occur in the eastern equatorial region of Ecuador and northern Peru. For example, the Sechura desert (4.5–7°S) receives up to 4 m of precipitation from December-June during EENE, while it receives almost no precipitation during the same period in normal years^47^.

### Sediment flux data analysis on the western Peruvian Andes

This study compiled an unpublished hydro-sedimentological dataset from 20 watersheds (Fig. 1). Hydrologic stations have basic equipment, such as limnigraphs/limnimeters, to record hourly water levels. Daily to monthly stream gauging has occurred since 1968 to monitor changes in the calibration curves and to calculate hourly water discharges. Daily average SSC samples are available from 1972 to 2012. Long-term data series include the EENE of 1982–1983 and 1997–1998 (Table 2), yet some hydro-sedimentological stations have no information during EENE (Supplementary Table 1). Nevertheless, SSC samples at each station include extreme water discharge peaks, those considered above 95^th^ percentile of the historic Q time series^48^ (see shaded areas in the Supplementary Fig. 2). Estimate of SSY uncertainty due to the infrequent sampling of SSC was based on Morera-Julca, *et al*.^49^. They indicate that a daily SSC sampling identifies annual and seasonal sediment flux cycles in the study area. Because the SSC dataset includes some temporal gaps, we rely our analysis on the use of sediment rating curve (SRC) to complete the time series (see Dataset and Data Analysis section).

**Table 2:** Quantitative analysis of the observed climatology, hydrology and fluvial sedimentology of the monitored watersheds.

Code	Climatology and Hydrology					Sedimentology																
	TRMM (1998–2012)	Runoff Without EENE	Runoff 1982–1983	Runoff 1997–1998	Water discharge	SSY (t/km^2^/yr)		Variant I			Variant II (lower segment)			Variant II (upper segment)			Variant III (lower segment)			Variant III (upper segment)		
	(mm/yr)	(l/km^2^/s)	(l/km^2^/s)	(l/km^2^/s)	(m^3^/s)	Without EENE	Historic	a	b	r^2^	a1	b1	r_1_^2^	a2	b2	r_2_^2^	c1	d1	r_1_^2^	c2	d2	r_2_^2^
1	671	13.2	81.5	—	11.7	347	763	3.10	0.96	0.43	5	−0.79	0.02	1.3	1.19	0.57	—	—	—	**11.1**	**0.69**	**0.61**
2	757	23.0	116.3	82.8	116.1	482	1941	6.18	0.77	0.43	40	0.13	0.01	1.6	1.05	0.44	61	0.07	0.01	**43.4**	**0.58**	**0.51**
3	1076	15.1	42.8	27.8	39.5	638	1027	10.30	0.93	0.46	60	0.24	0.02	**0.8**	**1.56**	**0.48**	102	0.19	0.05	0.5	1.34	0.41
4	629	9.5	42.2	48.7	137.5	309	1039	**6.42**	**0.84**	**0.50**	12	0.58	0.09	6	0.84	0.43	70	0.03	0.01	20.9	0.68	0.39
5	1058	23.8	52.6	53.7	107.8	499	—	—	—	—	—	—	—	—	—	—	—	—	—	—	—	—
6	896	8.8	35.7	42.5	135.4	11	162	3.23	0.48	0.20	7	0.28	0.03	**1.6**	**0.62**	**0.26**	20	0.05	0.01	6.7	0.42	0.25
7	781	5.1	34.4	28.8	100.9	260	431	2.99	0.98	0.68	232	−0.67	0.04	**2.5**	**1.02**	**0.68**	71	−0.08	0.01	30.9	0.68	0.62
8	679	6.0	33.4	38.2	34.1	732	1078	58.27	0.54	0.54	83	0.23	0.08	**4.7**	**1.16**	**0.70**	111	0.36	0.20	8.6	1.13	0.26
9	578	2.4	—	41.0	18.4	426	967	18.92	0.55	0.18	68	−0.02	0.01	**0.12**	**1.65**	**0.44**	110	−0.15	0.01	0.19	1.63	0.37
10	578	5.7	47.8	60.1	54.7	193	3069	7.80	1.01	0.66	29	0.59	0.06	**2.6**	**1.23**	**0.71**	197	−0.04	0.01	14.0	1.03	0.53
11	760	8.4	—	26.2	28.4	608	1100	10.45	0.84	0.63	22	0.33	0.16	**0.1**	**1.90**	**0.72**	40	0.11	0.03	17.3	1.01	0.56
12	699	7.2	18.0	23.5	26.8	404	2279	**0.43**	**1.75**	**0.50**	7	0.79	0.02	0.2	1.95	0.45	68	0.29	0.04	5.4	1.39	0.36
13	835	18.4	—	—	20.0	321	—	8.72	0.92	0.51	21	0.42	0.03	**6.5**	**1.00**	**0.43**	100	0.24	0.06	39.5	0.67	0.25
14	658	9.0	—	—	28.1	1925	—	32.19	1.20	0.75	87	0.80	0.14	**18.3**	**1.40**	**0.70**	607	0.18	0.03	561.0	0.75	0.61
15	1032	17.8	17.0	39.1	89.3	846	1181	0.13	1.75	0.74	1	1.18	0.07	**0.1**	**1.86**	**0.67**	81	0.12	0.01	8.1	1.22	0.61
16	835	13.0	17.7	21.5	140.0	1350	1659	0.56	1.47	0.78	44	0.37	0.02	**0.1**	**1.83**	**0.72**	23	0.06	0.01	30.0	0.98	0.51
17	914	15.9	—	—	114.1	594	—	0.48	1.38	0.71	5	0.78	0.23	**0.01**	**2.08**	**0.54**	93	0.04	0.01	23.1	0.85	0.62
18	638	8.2	8.5	13.3	50.1	137	203	3.79	1.15	0.29	21	0.53	0.02	1.2	1.41	0.12	152	0.25	0.04	**80.6**	**0.72**	**0.17**
19	620	5.3	—	9.4	106.0	148	419	0.01	1.94	0.58	120	−0.61	0.01	0.001	2.47	0.62	12	0.07	0.01	**7.9**	**0.94**	**0.67**
20	327	1.2	—	2.75	4.1	123	179	**90**	**1.30**	**0.52**	211	0.57	0.08	8.1	2.19	0.43	346	0.42	0.07	137.6	1.62	0.66

“Historic” indicates that the entire database was considered. “Without EENE” excludes the period 1 September 1982 to 31 August 1983 and 1 September 1997 to 31 August 1998. *a, a1, a2, c1, c2* and *b, b1, b2, d1, d2* are the constant and exponent in equations (1), (2) and (3) (see Dataset and Data Analysis). Bold number indicate the chosen SRC variant.

Distribution of monthly averaged SSC and Q for each station shows two marked patterns, one with a power-law univocal relationship and one with a clockwise hysteresis loop (Supplementary Fig. 1). Univocal SSC-Q relationships were found for mountain stations located downstream of low-slope alluvial plains with extensive sediment deposits. We suggest that these extended sediment deposit zones act as buffer zones accumulating sediments during dry phases and releasing them by re-incision^19^ during wet phases, especially during EENE (Supplementary Fig. 1 and Table 1). This is also supported by the general observation that univocal relationship occurs when the supply or availability of sediment is not the limiting factor^36^. At the contrary, clockwise hysteresis loops were observed for the SSC-Q relationship at piedmont stations. Several exceptions were found at stations downstream of reservoirs (stations 3, 7, 10, and 12). Clockwise hysteresis loops are generally interpreted by either a partial exhaustion of sediment availability during the last wet episodes^50^, and/or by a delayed water dilution effect in catchments due to contribution of tributaries or groundwater baseflow^51^. The larger the baseflow or dilution effect exists, the more pronounced the hysteresis is^52^. Annual bathymetric data (1976–2012) were analysed for the Poechos (88 m a.s.l), Gallito Ciego (380 m a.s.l) and El Platanal reservoirs (1531 m a.s.l) (Fig. 1) to quantify sediment yield. Six stations located downstream of the reservoirs were used to estimate seasonal and annual sediment fluxes. Stations 6 and 12 are located at the outlet of the reservoirs, while stations 3, 7, 10, and 18 are located more downstream near the Pacific Ocean (Fig. 1). For all these former stations, SSC records are not suitable for hydrological conditions during normal years because the reservoir acts as a barrier and captures a non-negligible portion of the total SSY upstream. However, their locations on the floodplain help us to understand the impact of inversed spatial rainfall distribution on desert areas during EENE.

## Results and Discussion

### Water and sediment fluxes during Extreme El Niño and normal years

**Figure 2:**
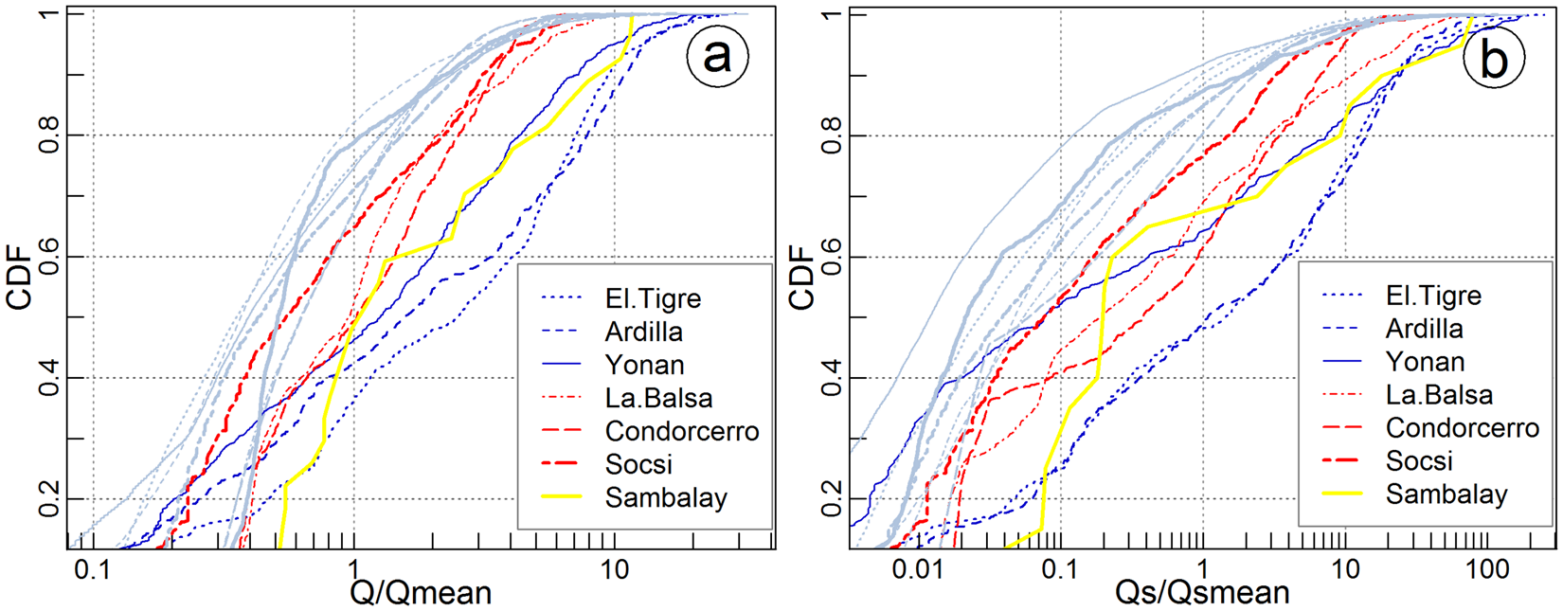
Normal-log plot of the Cumulative Density Function (CDF) of daily (**a**) water discharge and (**b**) suspended sediment flux (Qs) both normalized to historical average time series along the west central Andes. Grey lines were estimated from historic series without EENE periods; and coloureds lines were estimated just for EENE periods.

To characterize statistics of river hydrology during EENE and normal periods, we rely our analysis on the use of cumulated density function (CDF) of daily river discharge from long-term time series (Fig. 2a). We use the CDF to assess the impact of EENE on the frequency of high (i.e. *Q*/*Q*_*mean*_ > 1) and low (i.e. *Q*/*Q*_*mean*_ < 1) discharge events, as respect to the historical mean discharge. For all the catchments, the frequency of low discharge events follows similar behaviour, and does not change significantly when comparing normal with EENE periods. However, the frequency of high discharge events increases significantly during EENE. This increase is greater for catchments located in northern Peru (3–7°S, in blue Fig. 1c) than in central of southern Peru (7–18°S, in red Fig. 1c). Indeed, during normal periods about 90% of daily discharge events occur for *Q*/*Q*_*mean*_ < 2.5 for all the catchments, while during EENE periods, 90% of daily discharge events occur 1) for *Q*/*Q*_*mean*_ < 12 or 8 when considering the Ardilla and El Tigre stations (i.e. northern catchments), respectively; or 2) for *Q*/*Q*_*mean*_ < 4 when considering the Socsi, Condorcerro and La Balsa stations (i.e. central catchments).

Because of the obtained SRC, coefficient *a* and parameter *b*, relationship between water and sediment discharge, we expect that EENE influences sediment discharge as well. Therefore, we apply the same statistical method to assess the impact of EENE on river sediment discharge (Fig. 2b). For all the catchments, the frequency of low sediment discharge events follows similar behaviours, and does not change significantly when comparing normal with EENE periods. The frequency of high sediment discharge events is significantly impacted by EENE, with a higher proportion of large events, especially in northern Peru. Yet, even some stations located in central Peru (i.e. Socsi) also display a significant change in the frequency of high sediment discharge.

These results illustrate the variable impact of EENE on the hydrology and sediment transport of rivers in Peru, that is associated with more frequent high water and sediment discharge events, especially in northern Peru, without significant changes in the frequency of low discharge events. The geographic transition between these two hydrological and sedimentological regimes (northern vs central and southern Peru) is remarkably sharp (Supplementary Fig. 3) and differs from the transition in hydrological regime observed from 30–35°S in Chile^53^. In Chile, the transition in hydrological regime is also influenced by the contribution of EENE to the annual water discharge, which increases north from 35°S with aridity of the climate. In Peru, the contribution of extreme events to the water balance decreases southward with aridity, indicating a hydrological context different than those in Chile. High water discharge events (mean discharge plus 5 times the standard deviation) during normal years also appear south of 7°S, which indicates that the influence of local climate on water discharge is not yet clearly defined.

### Spatial impact of seismicity and precipitation on sediment transport during normal years

**Figure 3:**
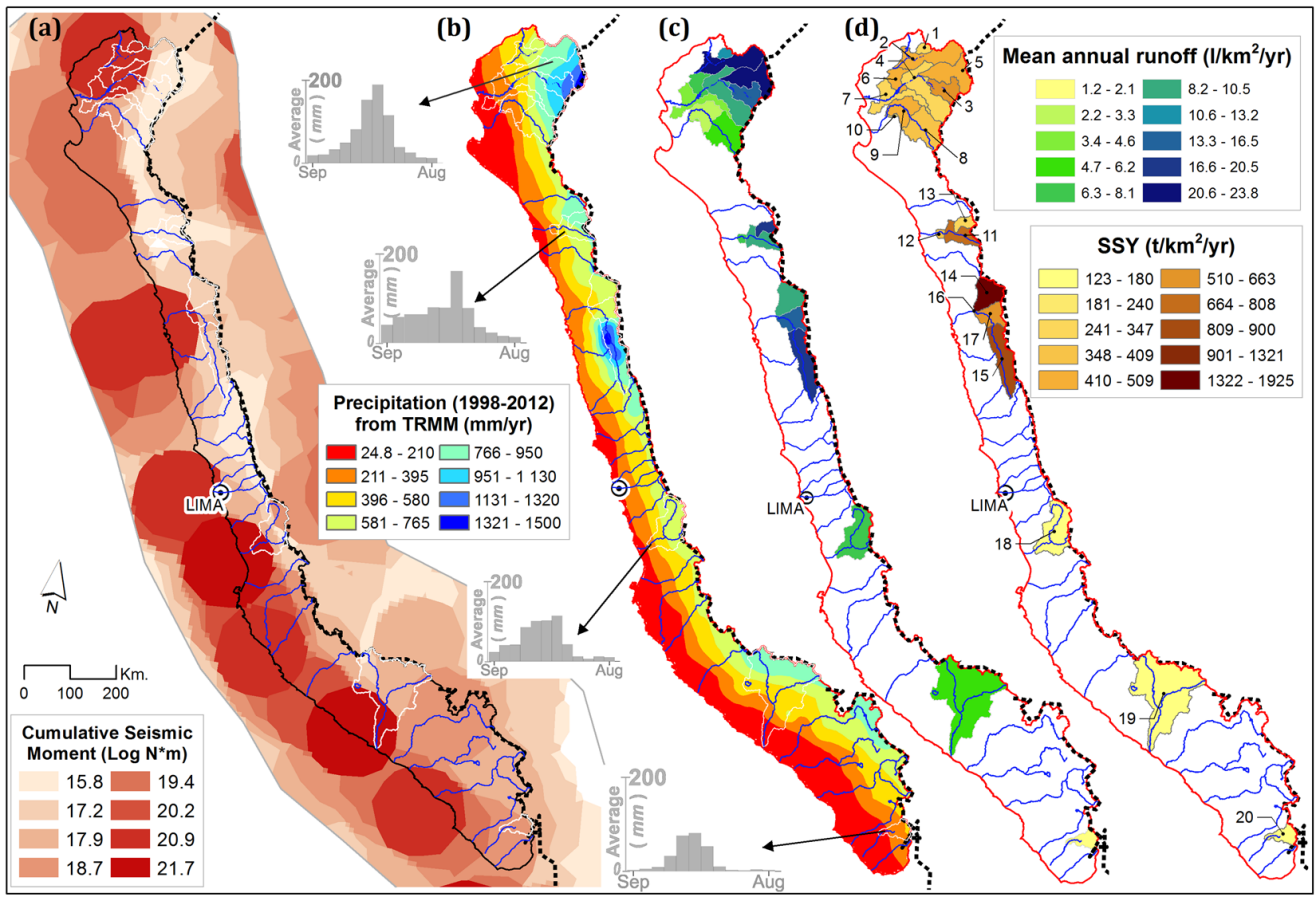
(**a**) Historical earthquake magnitude frequency within 50 km of each watershed. Brown circles represent the historical seismicity recorded by the USGS from 1900–2015. (**b**) Average spatial gradient of annual precipitation (mm/yr) from TRMM (3B43-V7) at 0.25° × 0.25° resolution (1 September 1998 to 31 August 2012). (**c**) Strong latitudinal and longitudinal gradient in mean annual runoff. (**d**) Suspended sediment yields (SSY) calculated from long-term series (without extreme El Niño events, 1 September 1982 to 31 August 1983 and 1 September 1997 to 31 August 1998). High SSY and random spatial variability are found along the Andes. The map was generated using ArcGIS for Desktop 9.3 (www.esri.com/software/arcgis).

To infer the potential environmental factors that could influence SSY during normal years, we analyse the spatial distribution of SSY relatively to mean annual precipitation and to seismicity, excluding EENE years. Indeed, major earthquakes can trigger numerous landslides, and the last major Peruvian earthquakes had profound impacts on the landscape^54^. Following Dadson, *et al*.^55^, we use the spatial distribution of the regional cumulative seismic moment as a proxy to infer the potential impact of historical seismicity (1962–2012) on the spatial distribution of sediment transport rates (see Methods). Although major (Mw >8) monitored earthquakes occurred mainly in central and southern Peru (8–17.5°S), there is no obvious spatial correlation between seismic moment and SSY (Fig. 3a and d). Indeed, cumulated seismic moment is mainly focused along the subduction interface, below the coast line, while SSY tends to be greater in the east, close the main water divide. This suggests that large-magnitude earthquakes were not primary factor influencing sediment production and transport efficiency at the regional scale in Peru during the monitored period. However, the potential long-term influence of large-magnitude earthquakes older than the timeframe of the seismic catalogue is completely unknown.

On the other hand, annual precipitation is estimated using the Tropical Rainfall Measuring Mission (TRMM) satellite data (see section Dataset and Data Analysis). During normal years, the TRMM data reveals a strong E-W precipitation gradient in the western Peruvian Andes (Fig. 3b). This gradient with low values near the Pacific Ocean (24.8–210 mm/yr) and high values on the areas of high relief (766–1500 mm/yr) is associated with orographic precipitation as well as the moisture sources from the Amazonian region^43^. This E-W annual precipitation gradient coincides well with the spatial distribution of both water runoff (ranging from ~1 to ~24 l/km^2^/s) and SSY (ranging from ~123 to ~2000 t/km^2^/yr). This correlation is observed at least for northern Peru, where the data have better spatial resolution. Nonetheless, we can contemplate that it also extends to central and southern Peru (watersheds 18–20), following the precipitation pattern. In addition, to this orographic effect, we also observe a N-S precipitation gradient as illustrated by the presence of some hotspots of more intense precipitation (>1000 mm/yr) in northern and central Peru. These precipitation hotspots also spatially coincide with hotspots of water runoff and SSY (watersheds 5, 15, 16, and 17; Fig. 3b–e). As a result, annual runoff and SSY in the studied stations have moderate to strong positive correlations (0.5 < r < 0.9; p-value > 0.05). Moderate correlations (0.5 < r < 0.75) are found in the Poechos (watersheds 6, 7 and 10), Gallito Ciego (watershed 12) and El Platanal reservoirs (watershed 18), as well as in areas impacted by mining and with lithology singularities (watershed 14)^32^. Overall, our results indicate that spatial variations of precipitation and runoff at the watershed scale mainly determines the spatial changes in SSY during normal years. Seismicity, lithological changes or anthropogenic disturbances, such as land use or reservoir, only act as second order factors at regional scale.

### Spatial impact of Extreme El Niño events on sediment transport

**Figure 4:**
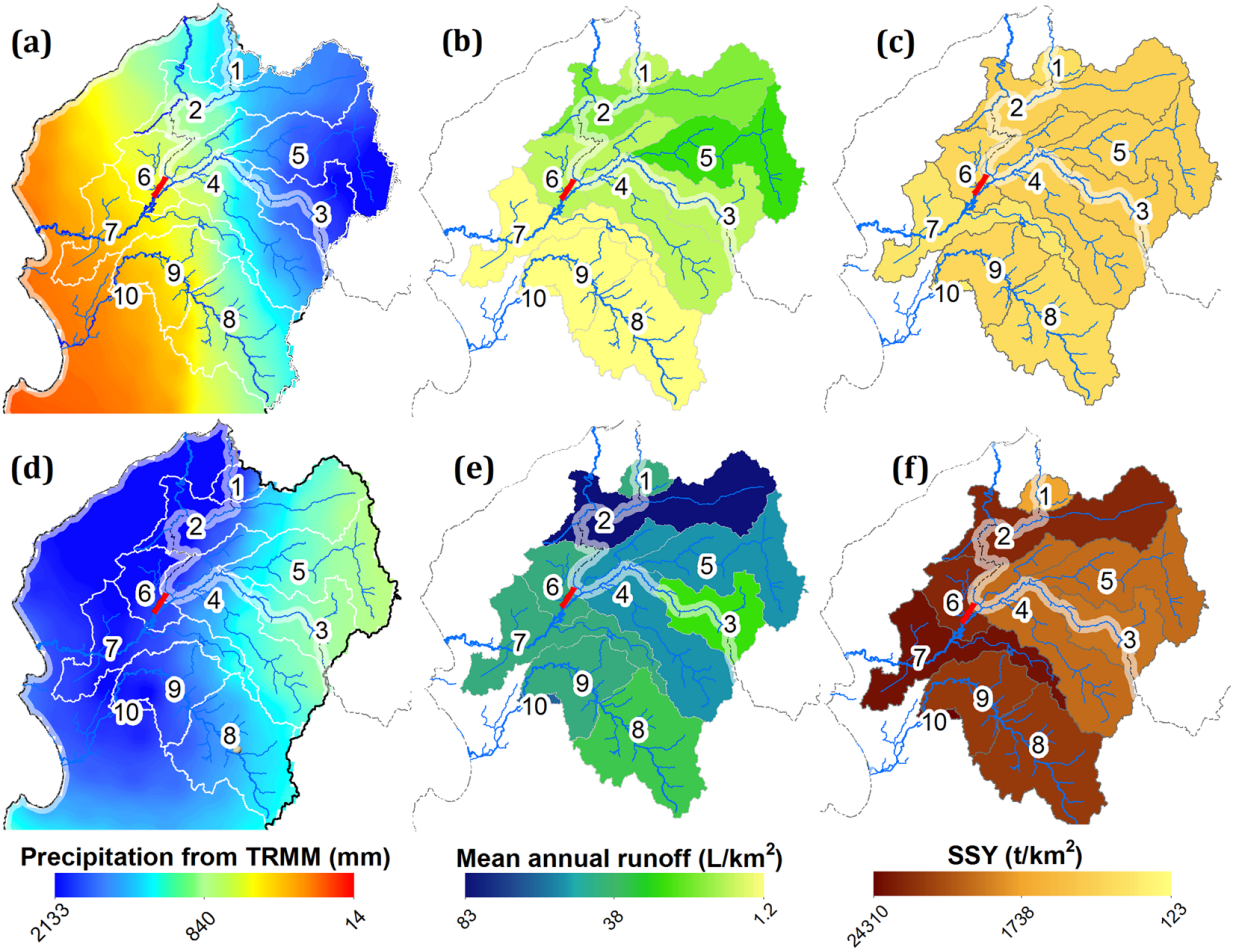
Close-up of 10 watersheds in the northwestern region from 3.4 to 5.7°S. (**a**–**c**) Show the mean cumulative precipitation amount, mean runoff and mean SSY, respectively, from January-April. All of them are plotted without the EENE period (January-April 1998). (**d**–**f**) Show the observed and estimated cumulative precipitation, runoff and SSY all during EENE (January to April 1998). Strong gradients are observed from east to west along the Andes. The red bar denoted the fixed weir at the outlet of the Venados river. The map was generated using ArcGIS for Desktop 9.3 (www.esri.com/software/arcgis).

**Figure 5:**
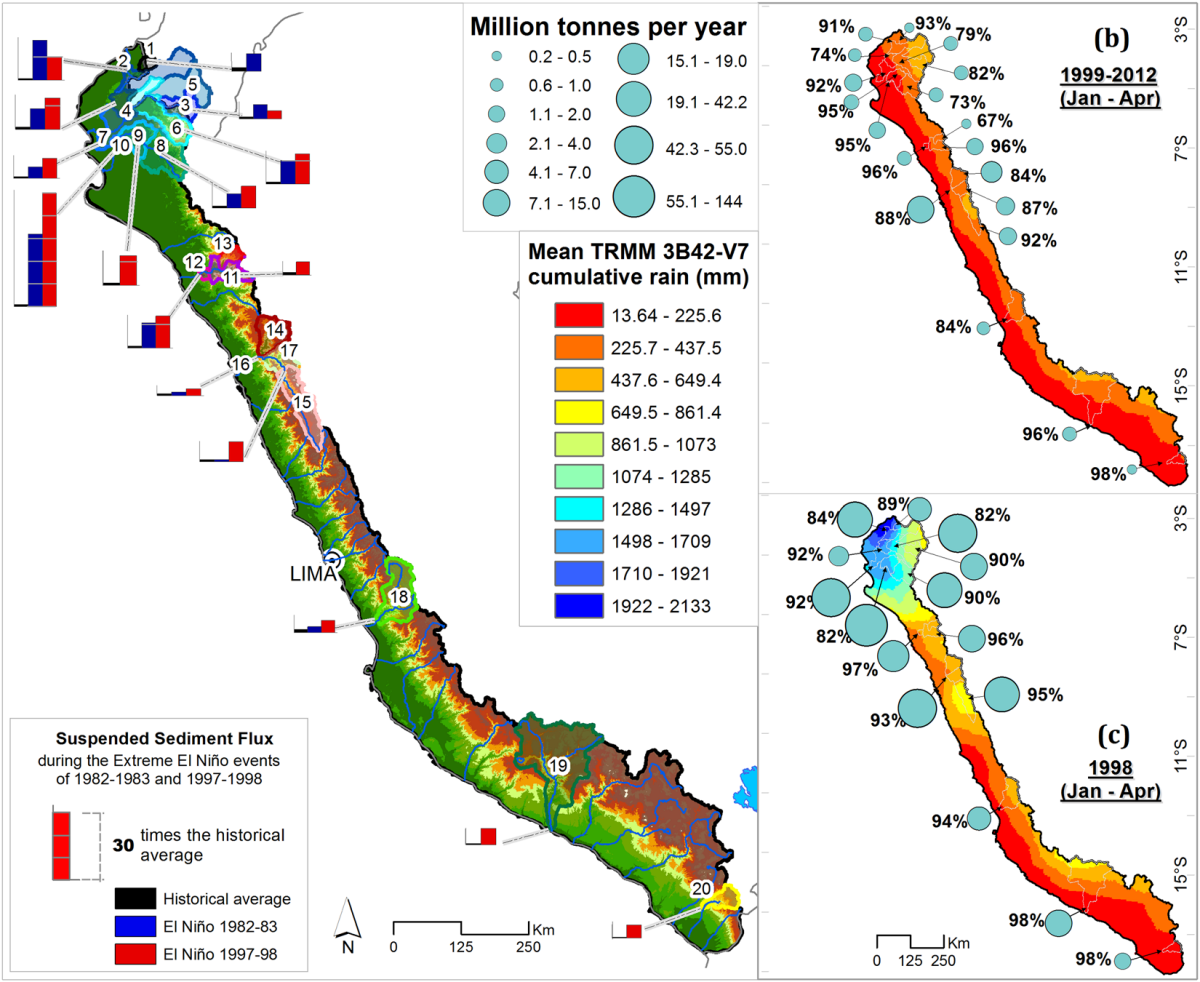
(**a**) Spatial distribution of suspended sediment flux during extreme El Niño events. Black bars represent historic sediment flux at each station, while red and blue bars represent sediment flux during the 1982–1983 and 1997–1998 extreme El Niño events, respectively. Each cube on the bar represents the sediment flux transported over 10 normal years. (**b**) Mean cumulative precipitation amount from January-April (1999–2012) without the EENE period (January-April 1998). Strong gradients are observed from East to West along the Andes. Circle size and the adjacent number indicate the sediment discharge and its percentage of annual SSY, respectively, from January-April (1999–2012). (**c**) Spatial precipitation patterns during the EENE period (January-April 1998). Circle size and the adjacent number indicate the sediment discharge and its percentage of annual SSY, respectively, from January-April 1998. The map was generated using ArcGIS for Desktop 9.3 (www.esri.com/software/arcgis).

The occurrence of EENE completely changes the intensity and spatial distribution of precipitation. Two regions with distinct precipitation patterns are observed during the wettest period of EENE (January-April): (i) central and southwestern Peru (7–18°S), where the normal spatial pattern of precipitation occurs, but with 2 to 4 times more precipitation than during normal years in the upstream part of the catchments (Figs 4 and 5c); and (ii) northern Peru (3–7°S), where the usual E-W spatial gradient of precipitation is inversed, with more precipitation in the west (Fig. 5c). During EENE, even dry areas experience high precipitation and runoff. Precipitation increases by 170-fold in the downstream part of the catchments located in North Peru (starting at the coast) (0–1100 m a.s.l.). From January to April, cumulative precipitation reaches 2133 mm, when it normally barely reaches 14 mm. As a result, usually dry to arid areas become the wettest part of the western Peruvian Andes during EENE. In the upstream part of the catchments of North Peru, cumulative precipitation in the wettest period increases by a factor 4 to 60.

We also observe a very strong increase in SSY during EENE for catchments located in north and central Peru (Fig. 5a–c). Usually dry-to-arid regions located in north Peru experience the strongest increase in SSY during EENE. We suggest these regions tend to accumulate sediment during normal years, due to low river transport capacity, and that the strong increase in precipitation and river transport capacity during EENE leads to high sediment discharge^56^. For example, the semi-arid areas between stations 6 and 7 (3403 km²), receive 2000 mm/yr of precipitation during EENE (height times larger than during normal years) and produces 26 Mt/yr and 44 Mt/yr of suspended sediment flux in 1982–83 and 1997–98, respectively, instead of 4,4 Mt/yr during normal year (Fig. 4). This is also supported by the observation that catchments in north and central Peru with a concave up hypsometric curve, associated to extended and relatively flat alluvial plains downstream, have a more pronounced response to EENE than catchments in the south with a concave down hypsometric curve, that display steeper and smaller alluvial plains downstream. This illustrates the likely role of the topography to modulate the sensitivity of outgoing sediment fluxes to climatic changes.

### Temporal impact of Extreme El Niño events on sediment transport

**Figure 6:**
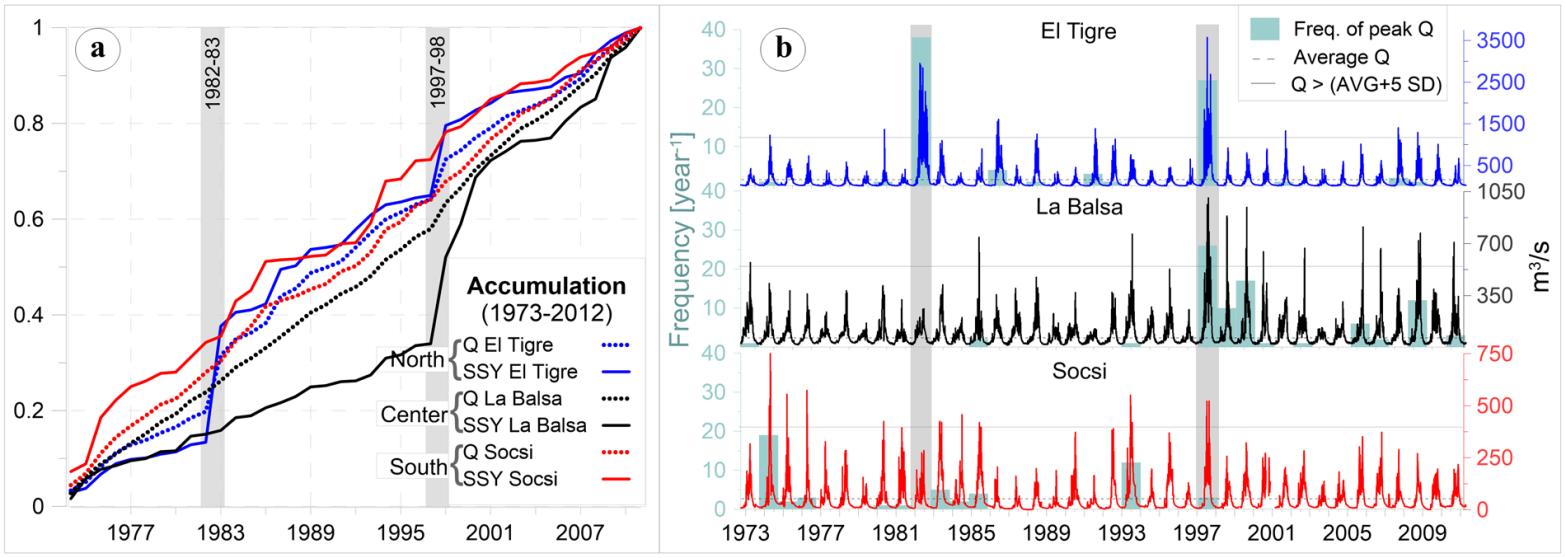
(**a**) Normalized annual suspended sediment yield (SSY) and water discharge (Q) plotted as cumulative annual values. Shaded bars indicate the two extreme El Niño events (1982–1983 and 1997–1998). (**b**) Daily Q series (m^3^/s) of rivers, from north to south. The dashed horizontal line represents Q mean at each station. The solid horizontal grey lines in each hydrogram mark the extreme Q peaks (mean discharge plus 5 times the standard deviation), while the blue bars indicate the number of times when Q exceeded it.

In agreement with Tarras-Wahlberg and Lane^37^, extreme rainfall and floods may occur in ENSO-neutral years and are related to annual oscillations in the InterTropical Convergence Zone (ITCZ). However, their assumption that other climate factors have more influence than EENE in causing above-average annual precipitation and floods in northern Peru is not verified in the historic Q series over the past 40 years (Fig. 6a,b). Cumulative water discharge in northern Peru increases linearly over time, with two sharp increases in 1982–1983 and 1997–1998 (Fig. 6a). Both altered the original progression of cumulative water discharge and contributed more than 20% of it over the 40 years (Fig. 6a). In contrast, cumulative water discharge in central and southern regions steadily increases, with multiple small breakpoints; one occurred in 1997–1998, but none was observed in 1982–1983 (Fig. 6a). Several extreme daily water discharges were observed from 1998–2000 in central Peru, and from 1974–1977, 1983–1986 and 1993–1994 in southern Peru, which seem influenced by local climate conditions that developed over days to weeks (Fig. 6b). Extreme rainfall and floods occurred during ENSO-neutral and La Niña years, but these events are isolated, differentiating from those observed during EENE.

Cumulative SSY in northern Peru shows two breakpoints, with sharp increases during EENE in 1982–1983 and 1997–1998 accounting for 45% of total cumulative SSY (Fig. 6a). Cumulative SSY has linear trends in central Peru, except during the second EENE, which transported 18% of 36 years of cumulative SSY. Frequent peak discharge transported 17% of the cumulative SSY over the next two years (Fig. 6a). This region seems highly sensitive to extreme meteorological events, with a 2-fold increase in mean annual water discharge generating a 7-fold increase in mean annual SSY. Ultimately, EENE is the main environmental factor influencing SSY in central Peru, even if other climatic events, such as those in 1998–1999 and 1999–2000, have also impacted SSY. In southern Peru, an irregular linear trend for the time evolution of accumulated SSY is observed with multiple breakpoints. One was influenced by the 1997–1998 EENE, however other local climatic events might also influence SSY in this region, see 1993–1994 which was classified as weak El Niño (http://ggweather.com/enso/oni.htm).

### Bed load transport and reservoir sedimentation during extreme El Niño events

During EENE, spatial and temporal SSY variability increases greatly, ranging from 3–60 times the historical annual average, with a strong N-S gradient (Fig. 6a). One hundred percent of mean annual SSY can be transported in less than 1% of the usual time. (Fig. 5a). Annual bathymetry data (1976–2012) at the Poechos reservoir indicates that 60% of its capacity was filled by sediment over the past 36 years. Most of the sediment load (63%) was transported during the two EENE, representing only 2.8% of the entire monitored time period. Poechos reservoir bathymetry data indicates that 75 Mt of total sediment was accumulated during the 1982–1983 EENE, including suspended and bedload sediments. However, this year 43 ± 6 Mt of suspended sediment was calculated at the Ardilla station (station 4), which is the main input of the Poechos reservoir (Fig. 5c and Supplementary Table 1). Around the Poechos reservoir, at the outlet of the non-perennial Venados river, a fixed weir was built (Fig. 4), which was completely filled with sediment after two normal years, with average precipitation of 424 mm/yr. During EENE, the precipitation increases to 2000 mm/yr, and the suspended sediment transport increase of 10–15 folds in catchments 6 and 7 (Table 2). We roughly estimate that about 12 Mt of sediment came from those arid areas (1611 km^2^) around the reservoir without transiting through the Ardilla station. This indicates that about 23 ± 7 Mt (19 to 40%) of the total accumulated sediment in the Poechos reservoir was transported as bedload. Hence, considering only suspended sediment and not the total sediment load to infer erosion rates during EENE would clearly lead to underestimated values of total load sediment transport. The quantification of bedload sediment transport is required to infer meaningful erosion rates.

Water supply and flood mitigation reservoirs are a major result of reservoir management, as well as reservoir sedimentation represent one of the main drivers of fluvial sediment transfer from watershed mountaintops to the ocean^57,58^. However, during EENE in northern Peru, high intensity rainfall events occurs in the lower part of the watershed near the coast. Therefore, the influence of reservoirs (constructed above 80 m a.s.l.) on the total sediment delivery ratio at the outlet of the catchment is partial.

## Conclusions

The impact of extreme El Niño events on sediment transport in the western Peruvian Andes (3–18°S) was investigated by compiling an extensive daily suspended sediment dataset (1968–2012) for 20 mountainous watersheds.

During normal years, the orographic effect of the Andes causes a strong gradient in precipitation, runoff (1.2–23.8 l/km^2^/yr) and SSY (123–2000 t/km^2^/yr). We observe that the spatial distribution of mean annual SSY is correlated to the spatial distribution of precipitation and water runoff. Although the Peruvian coast is in one of the most tectonically active regions in the world, the spatial distribution of historical seismic moment is not correlated to the spatial distribution of SSY, suggesting that earthquakes are not one of the primary factors influencing SSY at the regional scale.

During EENE (1982–1983 and 1997–1998), the intensity and spatial distributions of precipitation, runoff and precipitation change dramatically, in particular in the north of Peru. In North Peru, dry-to-arid areas, located downstream and close to the coastline, receive from 14 to 170 times the normal mean precipitation, while wet areas, located close to the main divide, receive up to 3 times the mean precipitation during normal years. Combined with the presence of extended flat alluvial plains downstream, that store sediments during dry conditions but release them during wet conditions. A 3- to 60-fold increase of SSY and an inversion of the spatial distribution of SSY occur during EENE years, with the downstream parts of the catchments contributing to most of the overall sediment discharge. In central and southern Peru, the response of sediment transport to EENE is less pronounced. We explain this by the absence of any inversion of the spatial distribution of precipitation during EENE and by the absence of extensive alluvial plains able to buffer sediment transport, as roughly illustrated by the concave down shape of the hypsometric curve of catchments in central and southern Peru.

Temporal variations of SSY show that SSY is more strongly influenced by infrequent EENE than by normal or moderate events. During EENE years, 82–97% of annual SSY occurs from January-April. During the same period, the amount of SSY cumulated during 3.5 days is equal to the total annual SSY in a normal year. Associated to suspended sediment transport, we also observe a sharp increase of bedload transport during EENE, as recorded by sedimentation in reservoirs.

An EENE has not occurred since August 1998. For nearly two decades (1998–2015), material eroded from upper portions of watersheds has been deposited on riverbanks and along hillslopes. Future EENE might transport more sediments than the last two EENE. A hydro-sedimentological monitoring network was set up in northwestern Peru to assess total sediment load during extreme floods and/or EENE. Future work will focus on the influence of suspended and bedload sediments on total sediment load during floods and/or EENE.

Overall, our study illustrates the influence of climate inter-annual variability and extreme climatic events that modulate river sediment transport and influence landscape evolution. With the current climatic conditions, the source to sink range and sediment transit time on the Pacific coast of Peru is controlled by high frequency climatic variability e.g.^59–61^. We also show that on a multidecadal time scale desert areas may have average erosion and sediment transport rates equivalent to those for temperate areas, due to cyclic large rainfall events. In addition, our study shows that extreme climatic events such as El Niño can periodically remobilize sediments that are usually deposited in the alluvial plain^62^, in turn limiting the potential buffering effect of foreland basins. However, this impact of extreme climatic events varies spatially and may likely depend on the steepness and hypsometric properties of impacted catchments. Our results also suggest that deciphering climatic conditions and variability from sedimentation archives requires a temporal resolution shorter than the classical recurrence time of cyclic extreme events. This questions the significance of using sedimentation archives with annual or pluri-annual resolution to detect the influence of climatic conditions on erosion and sediment transport rates.

## Dataset and Data Analysis

### Topographic data and hypsometric curves

Accurate topographic data are difficult to obtain for remote mountainous regions. Global digital elevation models (DEMs) provide a valuable source of information. The freely available 90 m DEM from the Shuttle Radar Topography Mission (SRTM-V4.1) was used to determine geomorphologic characteristics. Landscape morphology and the degree of fluvial dissection were characterized using hypsometric curve^63^. Normalized hypsometric curves were plotted as the relative area (*a*/*A*) vs. the relative elevation (*h*/*H*), where A is the total area, *a* is the area above *h*, *h* is the minimum elevation and *H* is the maximum elevation minus *h* (Fig. 1).

### Regional precipitation data

Sparse networks of meteorological stations and gaps in time-series data are common issues in the Peruvian Andes. This lack of information was addressed by using remote sensing precipitation products such as the Tropical Rainfall Measuring Mission (TRMM). Products at high spatial resolution and high temporal frequency for periods longer than 15 years can be obtained freely from the Goddard Earth Sciences Data and Information Services Center (http://disc.sci.gsfc.nasa.gov). We estimated average annual watershed precipitation from daily TRMM (3B42-V7) at 0.25° × 0.25° spatial resolution for the period 1 January 1998 to 31 August 2012.

### Earthquake database

Several high-magnitude earthquakes have occurred along the subduction zone of Peru over the past century, such as the Mw 8.2–8.4 great Peru earthquake in 2001 or the Mw 8.0 Pisco earthquake in 2007^10^. Large earthquakes can trigger many landslides, which in turn can have pronounced and prolonged impacts on landscape dynamics e.g.^8^. In this study, following Dadson, *et al*.^55^, we use the spatial distribution of the regional cumulative seismic moment as a proxy to infer the potential impact of earthquakes on the spatial distribution of erosion rates. The calculation uses the Advanced National Seismic System composite earthquake catalogue hosted by the Northern California Data Center^64^, which records earthquakes from 1962–2012 for the study area. A circular moving window with a 100 km search-radius is used to estimate the cumulative seismic moment of earthquakes.

To decrease the influence of deep earthquakes, which are less prone to trigger landslides, we consider only earthquakes shallower than 50 km. Since the magnitude of completeness is approximately 4.5 in the study area, we integrate only earthquakes with magnitudes higher than that to decrease statistical bias. This is not a major limitation, since earthquakes with lower magnitudes do not generally trigger landslides.

### Water discharge, suspended sediment concentration and sediment yield

This study compiled an inedited hydro-sedimentological dataset from 20 watersheds on the western Peruvian coast (Fig. 1), which was processed and homogenized (Table 1) using the freely available hydro-sedimentology software Hydraccess 4.6 (http://www.ore-hybam.org). Five multipurpose hydraulic projects and the SO-HYBAM monitor sediment yields along the Andes. Hydrologic stations have basic equipment, such as limnigraphs/limnimeters, to record hourly water levels. Daily to monthly stream gauging has occurred since 1948 to monitor changes in the calibration curves to calculate hourly discharges. At the 20 hydro-sedimentological stations, Suspended Sediment Concentration (SSC) was sampled at the same point near the edge and river surface into ~700 ml bottles. Distribution of SSC in the water column was assumed to be homogeneous due to the turbulent fluxes in the mountainous rivers. In the laboratory, each water-sample bottle was weighed and then filtered under a vacuum pump through a 0.45-µm diameter cellulose fiber filter that had been weighed, as was the empty bottle. The filter with sediments was heated in the oven at 105 °C for 24 hours and then dried and weighed to determine suspended sediment mass and SSC. SSC samples from SO-HYBAM were obtained directly using the associated protocol (http://www.ore-hybam.org/index.php/esl/Tecnicas/Muestras). Daily historic SSC samples are available from 1972–2012. Sediment flux discharge (Qs) is then simply obtained by multiplying SSC by Q, while suspended sediment yield is obtained by diving Qs by the surface area of the catchment at the gauging station.

### Suspended sediment rating curve using total and direct water discharge

Suspended sediment rating curve (SRC), established between measured SSC and their related Q, have been widely used to infer SSC. However, relationship between SSC and Q can often be very complex and strongly non-linear^65^.

We use SRC in two cases: (i) to fill daily SSC gaps if there were less than 80% of observed daily values per year, or (ii) to infer daily estimates of SSC during EENE periods only (Sep. 1982 to Aug. 1983 and Sep. 1997 to Aug. 1998), if observed SSC were not available. We did not expect to complete the entire SSC time-series during the study period (e.g. 50 years), because in most cases SSC records were concentrated in continuous several years (e.g. 6 years). Gap percentages of daily values were quantified for each year on the period that extends from 01 September year1 to 31 August year2.

In order to increase the accuracy of the infer SSC, three variants of the SRC were tested and then the best fit was kept:

Variant I. - SRC (see Equation 1 below)

If there are less than 40% of gaps over one year, we use an annual SRC model computed only from the daily data obtained during the same year; otherwise, we use a global SRC model computed from the pluri-annual daily dataset.

Variant II. - Piecewise SRC (see Equation 2 below)

The use of segmentation and truncation to better fit the trends of data highly can improve SSC estimations^66^. Here two continuous functions were fitted (lower and upper segments), consequently, two pre-factors, two exponents and two coefficients of determination (r^2^) were calculated. The breakpoint was defined in two steps:

(i) We divide our datasets in lower and upper discharge classes by locating a temporal breakpoint at the intersection of the median of the SSC and the median of the Q original values^66^, however this procedure rarely leads to the largest r^2^. The lower piecewise SRC segment comprises low water discharges and they influence up to 3 ± 2% of the annual SSY estimation. The rest 97 ± 2% of the annual SSY depends of the upper piecewise SRC segment. Therefore, the second segment will determine the prediction accuracy of the annual SSY.
(ii) Hence, in order to find the largest r^2^ on the upper SRC segment, we explore in systematic way changing up and down several times the upper segment range of the independent variable (Q), until finding the largest r^2^. Then the rest of the dataset will defined the lower segment. Lastly, the breakpoint was defined for the intersection of the lower and upper segment projection.

Variant III. - Direct runoff piecewise SRC (see Equation 3 bellow)

To remove the potential contribution of groundwater, the baseflow (Qb), was computed using the generic filtering method^67^. The direct runoff (Qd) was excluded from total Discharge (Q), Q = Qd + Qb. Then, the whole procedure in variant II was followed, however this time we use Qd instead of Q.

The different equations used for the three variants are:

1 $$SSC=a{{\rm{Q}}}^{b}$$

2 $$SSC=a1{{\rm{Q}}}^{b1}\,{\rm{for}}\,{\rm{Q}} < {\rm{\beta }},\,{\rm{and}}\,SSC=a2{{\rm{Q}}}^{b2}\,{\rm{for}}\,{\rm{Q}}\ge {\rm{\beta }}$$

3 $$SSC=c1{{\rm{Qd}}}^{d1}\,{\rm{for}}\,{\rm{Q}} < {\rm{\beta }},\,{\rm{and}}\,SSC=c2{{\rm{Qd}}}^{d2}\,{\rm{for}}\,{\rm{Q}}\ge {\rm{\beta }}$$

where a, a1, a2, c1, c2 and b, b1, b2, d1, d2 are the pre-factor and exponent, respectively, determined with a linear least-square fitting algorithm using daily Q or Qd in m^3^/s and daily SSC in mg/L; β is the breakpoint. Numbers one and two, next to rating parameters, correspond to the first and second piecewise segment (Table 2).

Finally, the best variant (I, II or III) was selected based on the quantification of the Root Mean Square Error between the observed SSY and inferred SSY (Table 2 and Supplementary Table 1).

SSC-Q hysteresis effect, at stations 1 and 2, was partly removed by using variant III, and considering only the direct discharge (Qd). However, at station 18 and 19 SRC clockwise hysteresis loops exhibit fluctuations and dispersion of the Qd-SSC. We assume that this may be attributed to reservoir sedimentation and narrow river floodplain areas, respectively. Unfortunately, the limited SSC dataset at both stations (Table 1) prevented us from exploring in more detail the causes of the clockwise hysteresis loops for those catchments.

Along the study area the, annual piecewise SRC, coefficient *a* and *b* are highly variable without unequivocal trends (increase or decrease), and there is no evolution of the slope SRC after and before the EENE (1982–83 and 1997–98). In general, piecewise regression model showed better fit than linear least-square fitting algorithm, see Table 2.

## Electronic supplementary material

Supplementary information
