# Supplementary material for: The impact of extreme El Niño events on modern sediment transport along the western Peruvian Andes (1968–2012)
Source: Sci Rep. 2017 Sep 25;7:11947. doi: 10.1038/s41598-017-12220-x (PMC5613030; doi:10.1038/s41598-017-12220-x)
Supplement: Supplementary file 1 — Supplementary information [file 41598_2017_12220_MOESM1_ESM.doc]

# The impact of extreme El Niño events on modern sediment transport along the western Peruvian Andes (1968-2012).

Sergio B. Morera^1,2,*^, Thomas Condom^3^, Alain Crave^4^, Philippe Steer^4^, and Jean L. Guyot^5^

^1^Instituto Geofísico del Perú, Lima, 15012, Perú.

^2^Universidad Nacional Mayor de San Marcos, Lima, 15081, Perú.

^3^Université de Grenoble Alpes, CNRS, IRD, IGE, F-38000 Grenoble, France.

^4^UMR 6118 Géosciences Rennes, Université Rennes 1, CNRS, Rennes, 35043, France.

^5^Université de Toulouse, CNRS, GET, IRD Lima, 15036, Perú.

* [sergiobaymorera@gmail.com](mailto:sergiobaymorera@gmail.com), +51 (01) 317-2300

**Supplementary information:**


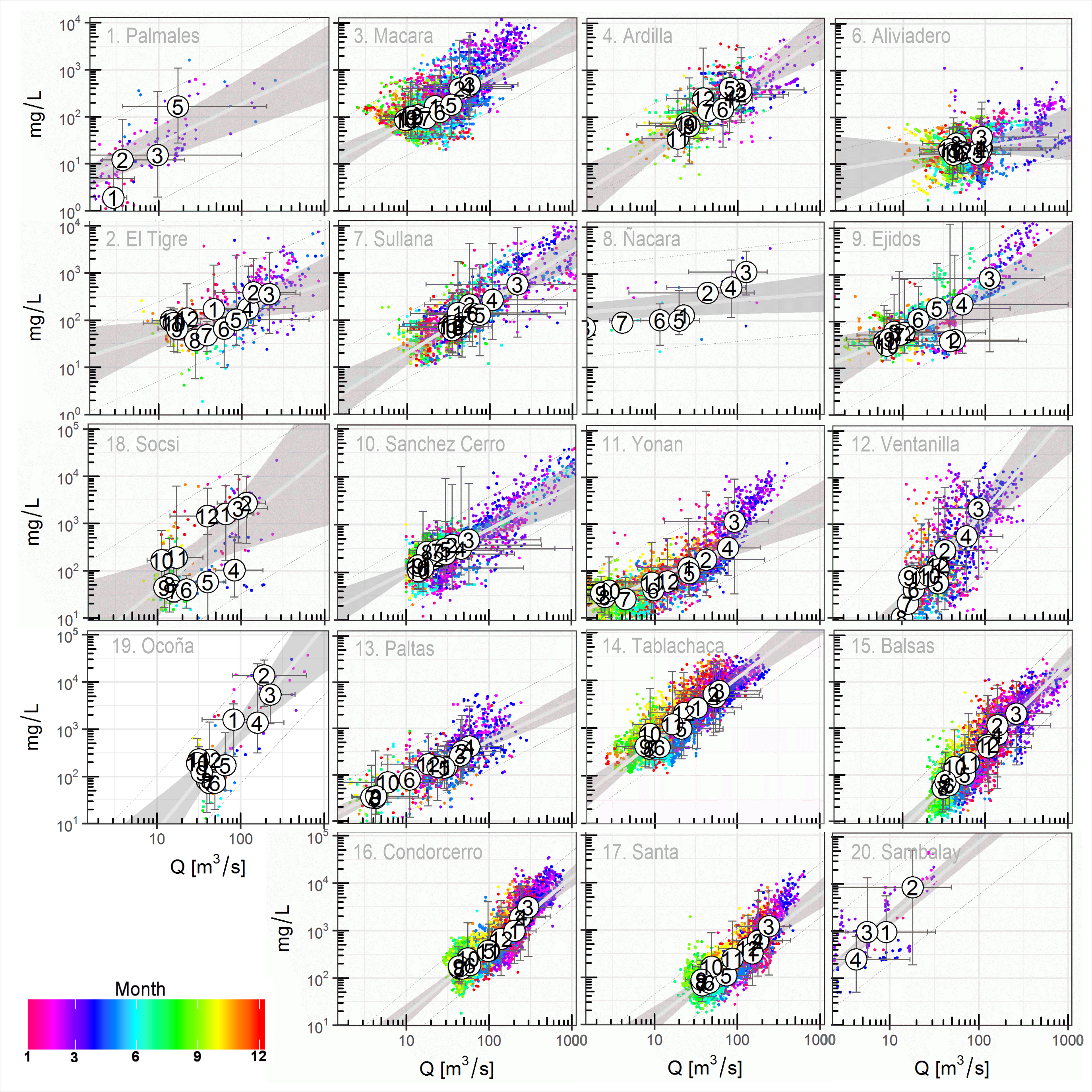


###### **Extended Figure 1.** Log-log plots of historical Sediment Rating Curves (SRC) between Suspended Sediment Concentration (SSC, in mg/L) and discharge (Q, in m3/s). Points are colored according to their measurement dates. Numbers in white circles indicate mean SSC and Q by month on the whole time-series, and error bars represent 5% and 95% quantiles from original values. The number before the station name corresponds to the code station indicated in Table 1. The light grey line is the regression of daily SSC and Q, while areas shaded in grey denote 10% and 90% confidence intervals of the original values. SSC, y-axis, at station 18 and 19 were multiplied by a factor of 10 to match the plot scale.


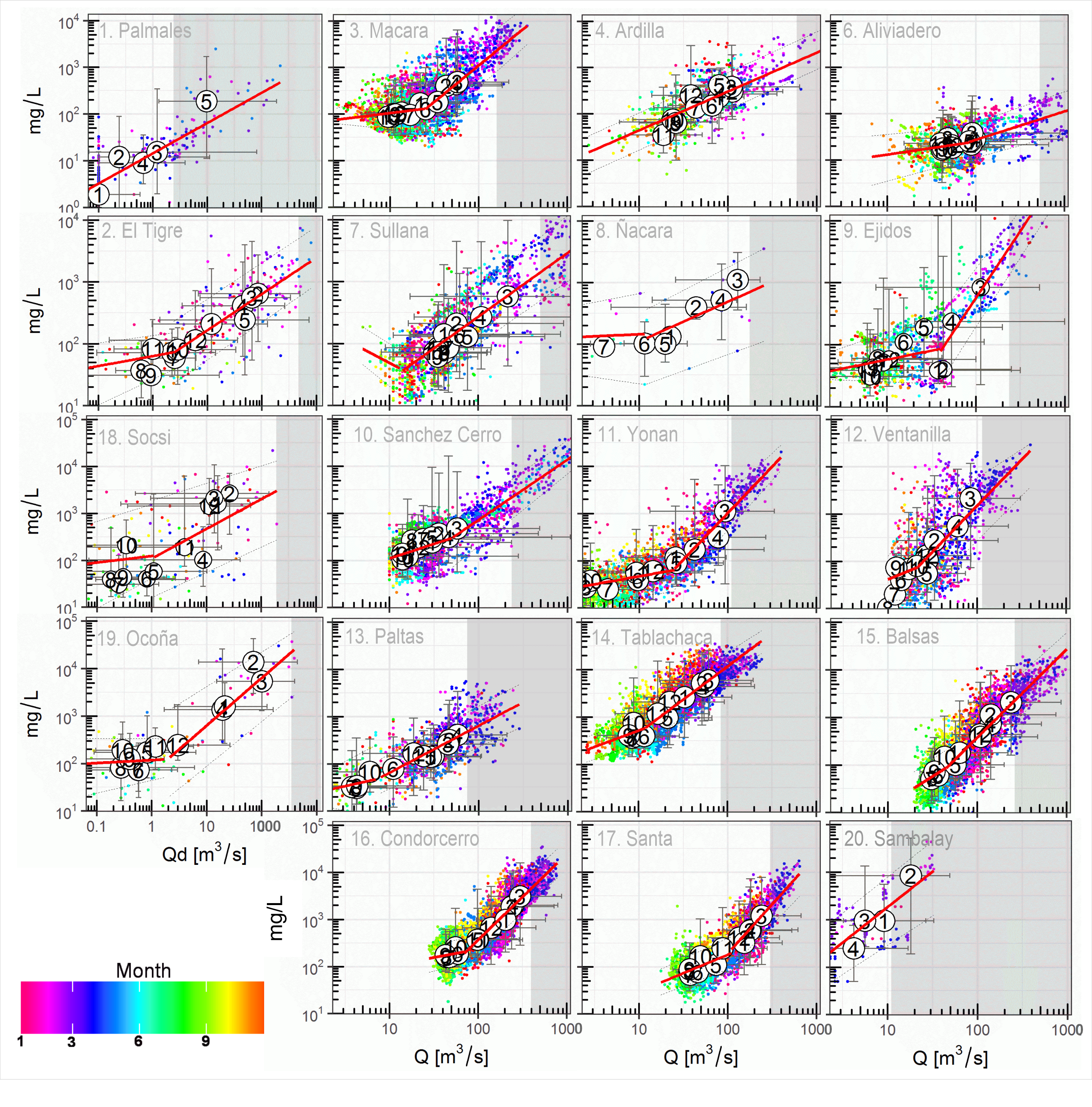


**Extended Figure 2.** Best fitted relationships between daily river discharges (Q) or direct daily river discharge (Qd) and daily suspended sediment concentration (SSC). Shaded areas, on the right side of each SRC graph, denote historical water discharge above 95th percentile of the natural values, which are consider extreme daily water discharge events . Perennial river discharge show two trends, one belonging to low water levels (dry season) and the other to the average and upper water levels (wet season), the upper trend slope is defined by the larger r^2^, and the lower trend is defined for the remaining dataset (SSC-Q). The intersection of both trends will locate the inflexion point. Unusually, Ardilla station shows one SSC-Q trend, the smoothing of the SSC during dry and wet seasons could be attributed to the Poechos reservoir operation. Temporary river discharge (stations 1 and 2) show just one trend (wet season). SSC, y-axis, at station 18 and 19 were multiplied by a factor of 10 to match the plot scale.


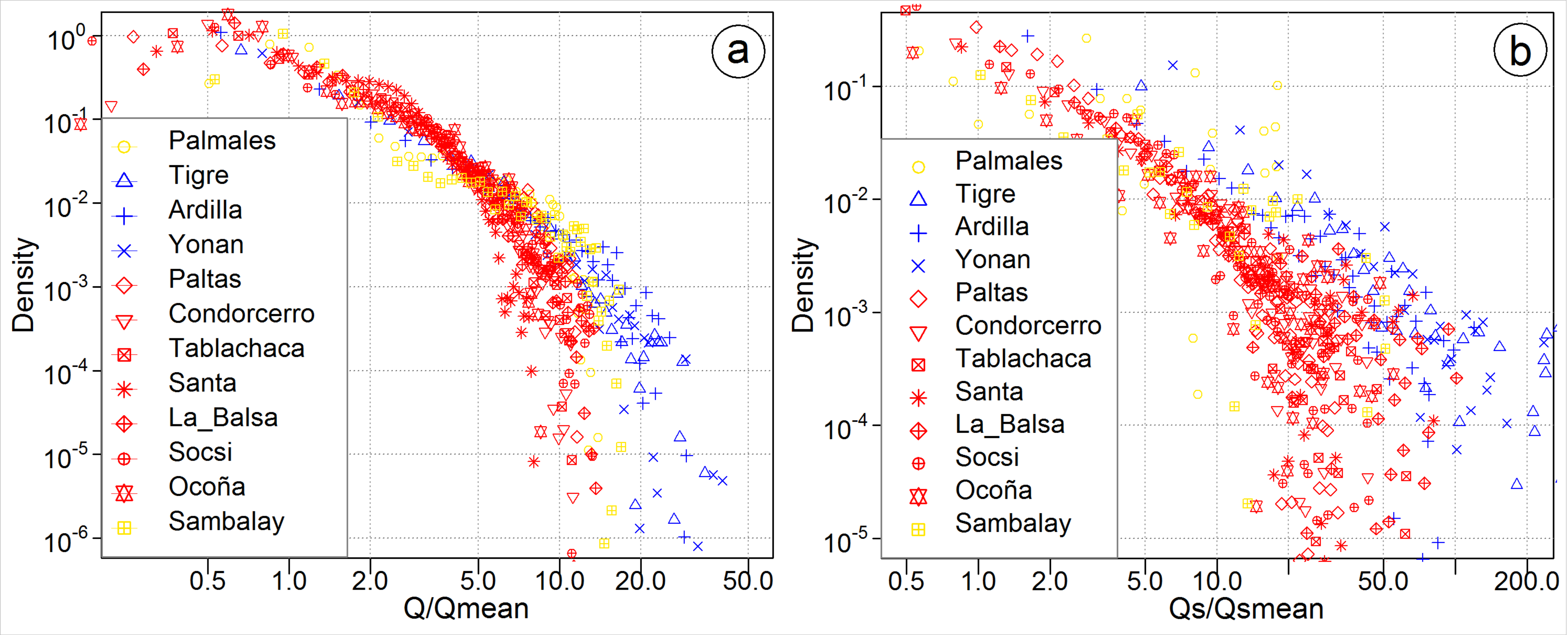


**Extended Figure 3.** Bi-log plot of the standardized PDF of a) daily water discharge (Q) and b) daily suspended sediment flux (Qs) during historical time series along the west central Andes.

**Extended Table 1.** Sediment Yield uncertainty analysis for all the catchments. Note that the goodness-of-fit metrics are all based on the mean daily flows for the historic period.

| Stations | Number of SSY samples (n) | | | SY Gaps percentage/yr | | | Predicted SY (Mt.yr^-1^) | | | Root Mean Square Error (Mt.yr^-1^) | | |
| --- | --- | --- | --- | --- | --- | --- | --- | --- | --- | --- | --- | --- |
| Code | No EENE | EENE (1982-83) | EENE (1997-98) | No EENE | EENE (1982-83) | EENE (1997-98) | No EENE | EENE (1982-83) | EENE (1997-98) | No EENE | EENE (1982-83) | EENE (1997-98) |
|  |  |  |  | % | % | % | Avg. | Avg. | Avg. | Avg. | Avg. | Avg. |
| 1 | 245 | - - - - | - - - - | 71 | - - - - | - - - - | 0.2 | 1.1 | - - - - | 0.14 | - - - - | - - - - |
| 2 | 2082 | - - - - | - - - - | 13 | - - - - | - - - - | 2.3 | 44.7 | 25.9 | 0.47 | - - - - | - - - - |
| 3 | 5624 | 272 | - - - - | 14 | 25 | - - - - | 1.6 | 11.5 | 6.6 | 0.33 | 1.70 | - - - - |
| 4 | 1804 | 122 | - - - - | 52 | 67 | - - - - | 3.7 | 43.0 | 54.5 | 1.11 | 5.70 | - - - - |
| 5 | - - - - | - - - - | - - - - | - - - - - | - - - - | - - - - | - - - - | - - - - | - - - - | - - - - | - - - - | - - - - |
| 6 | 3775 | 180 | - - - - | 10 | 51 | - - - - | 0.2 | 1.7 | 2.7 | 0.11 | 0.02 | - - - - |
| 7 | 6884 | 210 | - - - - | 13 | 42 | - - - - | 4.4 | 25.8 | 44.4 | 0.73 | 3.50 | - - - - |
| 8 | 19 | - - - - | - - - - | 99 | - - - - | - - - - | 1.8 | 23.6 | 36 | 0.40 | - - - - | - - - - |
| 9 | 5904 | - - - - | 232 | 15 | - - - - | 36 | 3.2 | - - - - | 46.3 | 1.14 | - - - - | 5.80 |
| 10 | 5252 | 210 | - - - - | 21 | 42 | - - - - | 1.5 | 144 | 226 | 1.83 | 20.70 | - - - - |
| 11 | 2964 | - - - - | - - - - | 10 | - - - - | - - - - | 2.2 | - - - - | 11.8 | 0.19 | - - - - | - - - - |
| 12 | 2556 | - - - - | - - - - | 12 | - - - - | - - - - | 1.5 | 18.7 | 23.7 | 0.21 | - - - - | - - - - |
| 13 | 1673 | - - - - | - - - - | 24 | - - - - | - - - - | 0.3 | - - - - | - - - - | 0.04 | - - - - | - - - - |
| 14 | 3542 | - - - - | - - - - | 12 | - - - - | - - - - | 6.0 | - - - - | - - - - | 0.38 | - - - - | - - - - |
| 15 | 7902 | 65 | 215 | 30 | 82 | 41 | 4.1 | 3.7 | 40.4 | 1.26 | 0.20 | 7.70 |
| 16 | 4726 | - - - - | - - - - | 9 | - - - - | - - - - | 14.1 | 24.1 | 47.7 | 0.71 | - - - - | - - - - |
| 17 | 3652 | - - - - | - - - - | 11 | - - - - | - - - - | 4.3 | - - - - | - - - - | 0.37 | - - - - | - - - - |
| 18 | 182 | - - - - | - - - - | 93 | - - - - | - - - - | 0.8 | 1.2 | 4.2 | 0.14 | - - - - | - - - - |
| 19 | 126 | - - - - | - - - - | 91 | - - - - | - - - - | 2.4 | - - - - | 24.1 | 0.29 | - - - - | - - - - |
| 20 | 419 | - - - - | 20 | 82 | - - - - | 95 | 0.2 | - - - - | 1.1 | 0.38 | - - - - | 2.20 |

**Reference:**

Seneviratne, S. I., Nicholls, N., Easterling, D., Goodess, C. M., Kanae, S., Kossin, J., Luo, Y., Marengo, J., McInnes, K., Rahimi, M., Reichstein, M., Sorteberg, A., Vera, C., and Zhang, X., 2012, Changes in climate extremes and their impacts on the natural physical environment*,* in Field, C. B., Barros, V., Stocker, T. F., Qin, D., Dokken, D. J., Ebi, K. L., Mastrandrea, M. D., Mach, K. J., Plattner, G.-K., Allen, D. J., Tignor, M., and Midgley, P. M., eds., Managing the Risks of Extreme Events and Disasters to Advance Climate Change Adaptation: Cambridge, UK and New York, NY, USA, Cambridge University Press, p. 109-230.
